# Impact of Xanthylium Derivatives on the Color of White Wine

**DOI:** 10.3390/molecules22081376

**Published:** 2017-08-19

**Authors:** Franziska Bührle, Anita Gohl, Fabian Weber

**Affiliations:** Institute of Nutritional and Food Sciences, Molecular Food Technology, University of Bonn, Römerstraße 164, 53117 Bonn, Germany; f.buehrle@uni-bonn.de (F.B.); s7angohl@uni-bonn.de (A.G.)

**Keywords:** xanthylium derivatives, white wine oxidation, color

## Abstract

Xanthylium derivatives are yellow to orange pigments with a glyoxylic acid bridge formed by dimerization of flavanols, which are built by oxidative cleavage of tartaric acid. Although their structure and formation under wine-like conditions are well established, knowledge about their color properties and their occurrence and importance in wine is deficient. Xanthylium cations and their corresponding esters were synthesized in a model wine solution and isolated via high-performance countercurrent chromatography (HPCCC) and solid phase extraction (SPE). A Three-Alternative-Forced-Choice (3-AFC) test was applied to reveal the color perception threshold of the isolated compounds in white wine. Their presence and color impact was assessed in 70 different wines (58 white and 12 rosé wines) by UHPLC-DAD-ESI-MS^n^ and the storage stability in wine was determined. The thresholds in young Riesling wine were 0.57 mg/L (cations), 1.04 mg/L (esters) and 0.67 mg/L (1:1 (*w*/*w*) mixture), respectively. The low thresholds suggest a possible impact on white wine color, but concentrations in wines were below the threshold. The stability study showed the degradation of the compounds during storage under several conditions. Despite the low perception threshold, xanthylium derivatives might have no direct impact on white wine color, but might play a role in color formation as intermediate products in polymerization and browning.

## 1. Introduction

**Figure 1:**
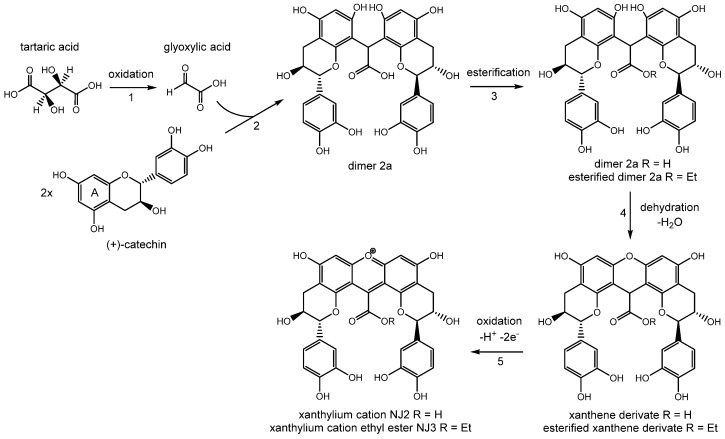
Mechanism of the xanthylium cation (NJ2) formation and its corresponding ethyl ester (NJ3) from (+)-catechin and glyoxylic acid via a colorless carboxymethine-linked dimer (dimer **2a**) and colorless xanthene [18].

White wine might develop various defects during storage and aging, including the loss of characteristic aromas and undesirable color changes. Among the different types of color deterioration, browning is the most frequently occurring phenomenon [1,2,3,4]. Despite the economic relevance, current knowledge about the related reaction mechanisms and the complex product profiles that cause discoloration is insufficient [5]. The oxidation of polyphenols involving either enzymatic or non-enzymatic reactions is generally considered to be the major browning process. The oxidation leads to the formation of quinones, which take part in polymerization processes. Flavanols like (+)-catechin and (−)-epicatechin are also related to color alterations in wines [6,7,8,9]. These alterations are mostly based on the reactions of flavanols with non-phenolic compounds like aldehydes [5,10,11,12]. Glyoxylic acid, an oxidized tartaric acid residue, contains an aldehyde group and is formed under oxidative wine storage conditions [13,14]. The presence of metal ions promotes the oxidative reactions [15,16,17]. In model wine systems, catechin has been shown to form yellow pigments in the presence of glyoxylic acid. These pigments have been identified as xanthylium derivatives [18,19]. The formation of xanthylium derivatives is based on a multistage sequence of reactions including a condensation reaction between catechin and glyoxylic acid. Figure 1 shows the reaction of the two compounds to form the so-called xanthylium cation NJ2 and its corresponding ethyl ester (NJ3) via the formation of a colorless carboxymethine-linked catechin dimer (dimer 2a, 8-8 bond) and a xanthene derivative [18]. Step three (esterification) is only required for the formation of esterified xanthylium cations and is omitted if applicable.

**Figure 2:**
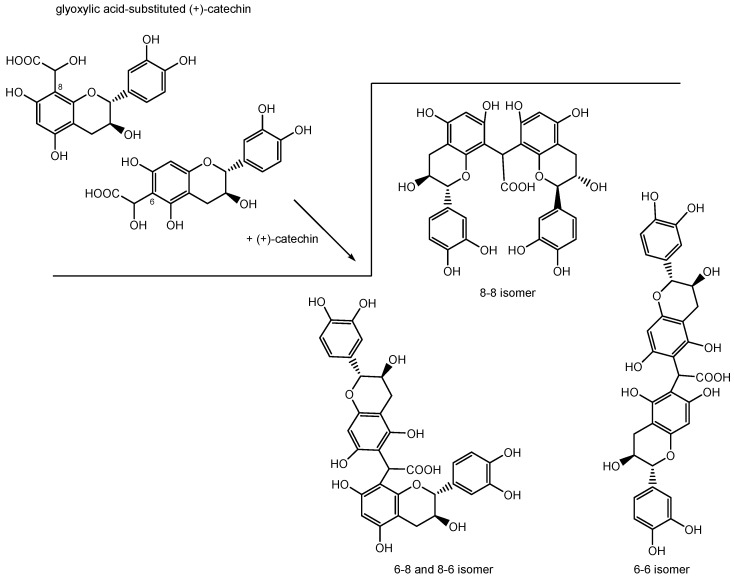
Formation of the three different carboxymethine-linked (+)-catechin dimers from (+)-catechin substituted with glyoxylic acid at ring position 6 or 8 [10,17,18].

Two other carboxymethine-linked dimers can be formed, due to the structure of the A-ring of catechin, whereby the dimers constitute a 6-6 and a 6-8/8-6 bond, respectively (see Figure 2) [5,10,17,18]. Consequently, six structural isomers of xanthene derivatives, xanthylium cations and their corresponding ethyl esters have been characterized, respectively [10,17,18,20]. One xanthene and subsequently one xanthylium cation derives from the 8-8 isomer, two from the 6-8/8-6 isomer and three from the 6-6 isomer, respectively [10,17]. The formation of xanthylium derivatives is influenced by various factors. Acidic conditions (pH 3.2) enhance the formation of xanthylium derivatives [21]. Protonated glyoxylic acid promotes phenolic polymerization reactions [13] and a high incubation temperature (45 °C) increases the reaction rate, as shown in earlier studies [17,22]. Copper and iron ions catalyze the reaction between catechin and glyoxylic acid [8,17,22]. Copper ions, which have a more pronounced effect on the formation of xanthylium derivatives than iron ions, promote the formation of dimers bound to the C-8 position, whereas ferrous and ferric ions enhance the formation of dimers bound via the C-6 ring position. Thus, metal ions influence the configuration of the resulting derivatives in wine-like and equimolar concentrations by forming different complexes with catechin and promote the oxidation reactions [17]. The UV-vis spectra of xanthylium cations show an absorbance maximum at 440 nm. The corresponding esters have an absorbance maximum at 460 nm. This gives rise to a yellow color for the cations and to an orange color for the esterified compounds [8,18,23]. Xanthylium-derived pigments have been detected in a red wine fraction [10] and a catechin-spiked white wine stored under sunlight exposure conditions [24] but have not yet been quantified in commercial white wines. It was observed that the presence of caffeic acid lowers the stability of xanthylium derivatives and interferes with the formation reaction, finally leading to the development of a brown color in model wines. Besides this observation, the instability of xanthylium cations under light exposure was shown [23]. The formation of yellow xanthylium derivatives in wine-like model solutions suggests a possible contribution in color evolution and browning during aging of white wine [5,18]. This assumption still needs to be confirmed. The aim of the present study was to investigate the role of xanthylium derivatives as pigments in white wine and to assess the color activity of these compounds. This work considers the role of xanthylium derivatives regarding the color formation and their possible influence on color defects in white wine.

## 2. Results and Discussion

### 2.1. Synthesis and Isolation of Xanthylium Derivatives

The reaction conditions for the controlled formation of xanthylium derivatives were adapted from George et al. [23] and were optimized in preliminary tests. Optimal precursor ratio (2:1 (+)-catechin/glyoxylic acid mole ratio), pH (3.2), temperature (45 °C), and the use of catalyzing metal ions (0.60 mg/L copper(II) and 1.50 mg/L iron(II)) led to a high formation of xanthylium derivatives in the wine-like medium. The formation of xanthylium derivatives was accompanied by a change in color of the model wine solution from slightly yellow to dark orange-red. In the present study, the amount of formed xanthylium derivatives was 35% of the potential amount regarding the catechin concentration. An average decrease of (+)-catechin in the synthesis mixture of 370 mg/L evoked a xanthylium derivative concentration of 98 mg/L (calculated as NJ2 equivalent) in average highlighting the broad spectrum of side-reactions. The by-products account for approximately 15% of the depleted catechin content.

#### 2.1.1. UHPLC-DAD-ESI-MS^n^ Identification of Xanthylium Derivatives

**Figure 3:**
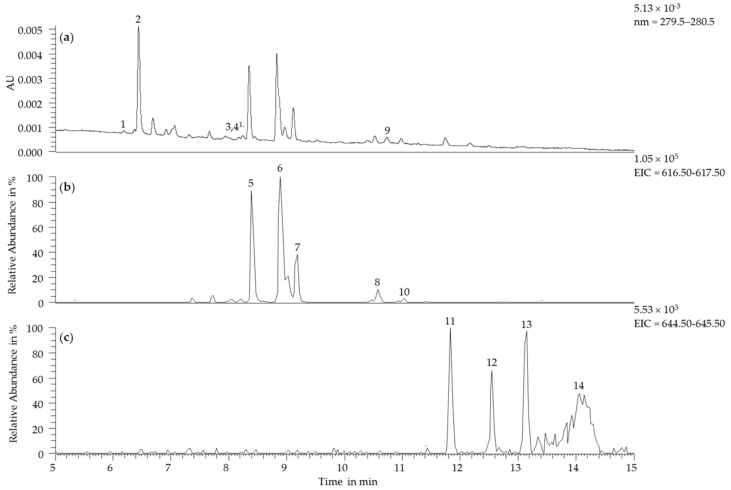
UHPLC-DAD-ESI^+^-MS chromatogram of the synthesis medium after an incubation of 15 days at 45 °C for the identified compounds (**a**) (+)-catechin (peak 2), carboxymethine-linked (+)-catechin dimers (peak 1, 3 and 4), xanthylium lactone (peak 9) at 280 nm; (**b**) non-esterified xanthylium cations at *m/z* 617 (peak 5−8, 10); (**c**) xanthylium cation ethyl esters at *m/z* 645 (peak 11−14). ^1.^ Peaks 3 and 4 were not detectable at UV-spectrum.

Analysis by UHPLC-DAD-ESI-MS^n^ was applied to investigate the chemical constituents of the reaction mixture. In total, 14 different compounds were identified in the reaction mixture. Besides five non-esterified xanthylium cations and four xanthylium cation ethyl esters, three carboxymethine-linked (+)-catechin dimers, one xanthylium lactone, and residual (+)-catechin were identified by UHPLC-DAD-ESI-MS^n^ analysis in accordance with previous reports [10,17,18,22,25]. The presence of residual (+)-catechin may be explained by the ongoing formation of xanthylium derivatives in the reaction mixture. Figure 3 shows the UHPLC-DAD-MS separation of all identified compounds (see Table 1) after 15 days of incubation. 

Based on the results reported by Es-Safi et al. [18], peaks 1, 3 and 4 were identified as carboxymethine-linked catechin dimers. The [M + H]^+^ ions at *m/z* 637 and further fragments at *m/z* 347 (−290 Da, loss of (+)-catechin moiety) and *m/z* 291 (−346 Da) reflect the dimeric structure. The product ion at *m/z* 485 arises from a Retro-Diels-Alder fragmentation [13,18,26]. The order of elution and the fragmentation patterns imply the identity of three carboxymethine-linked dimers [18]. The first dimer has a slightly polar character and elutes before catechin. It was tentatively identified as dimer **2a** [10]. The dimers **2** and **3** were tentatively identified as the 6-8/8-6 and 6-6 carboxymethine-linked catechin dimer isomers [17,22]. Regarding the non-esterified xanthylium cations, five out of six known derivatives have been detected (peaks 5−8 and 10). All xanthylium cation peaks produced product ions at *m/z* 465 and *m/z* 599. These ions correspond to the Retro-Diels-Alder fission with a loss of C_8_H_11_O_3_ and H_2_O, respectively. The product ions at *m/z* 447, *m/z* 421 and *m/z* 313 in the MS³ experiment were identified according to Labrouche et al. [25]. Due to its high abundance, cation 2 (peak 6) was tentatively identified as NJ2, which is derived from dimer **2a** [10]. Based on the MS³ data, the other four cations could not be assigned to their corresponding dimer or xanthene, respectively. Peak 9 (t_R_ = 10.8 min; *m/z* 619) was tentatively identified as xanthylium lactone. Xanthene derivatives as xanthylium derivative precursors show the same *m/z* as xanthylium lactones (*m/z* 319) and are preferably formed [10,17,22]. The fragment ion at *m/z* 327 corresponds to a loss of catechin from the lactone form and cannot be formed based on the xanthene form. This leads to the suggestion that compound **9** is a lactone derivative [17]. Peaks 11 to 14 showed product ions at *m/z* 645. This corresponds to the mass of esterified xanthylium cations. All compounds produced the same fragment ions, whereby the fragment ion at *m/z* 493 was attributed to the species-generic loss of 152 Da by Retro-Diels-Alder reaction [25]. Based on the elution order and concentration ratio, xanthylium cation ethyl ester 2 (peak 13) was tentatively identified as NJ3 [10]. Peak 14 eluted within a poorly separated hump of undefined compounds at the end of the chromatogram. A recent study by Guo et al. also reported co-elution effects for xanthylium cation ethyl esters [17]. Among the xanthylium derivatives, the fragment ion at *m/z* 447 (MS³) was the most abundant. This ion is generated by the dehydration of the (possibly esterified) carboxymethine bridge. Peaks that remained unidentified, such as a co-eluting peak occurring at 11.7 min (*m/z* 653 → 635 → 547, 591, 529, 441, 503, 617), might be ascribed to unknown oxidation products of (+)-catechin or other by-products of the formation reaction [10]. Besides the previously discussed compounds, catechin-trimers, xanthene-xanthylium trimers, xanthene quinone and xanthylium quinone have been described in the literature [5,9,17,27,28] but were not detected in the present study.

#### 2.1.2. High-performance Countercurrent Chromatography (HPCCC)

CCC is a liquid-liquid partition chromatographic technique which requires a high retention of the stationary phase in the column for high peak resolution. In an ideal separation, the mobile phase passes the system, while more than 50% of the stationary phase is retained. This can be verified by the determination of the separation time of the two phases. In general, it should not exceed 20 s. Extended settling times would reduce the retention of the stationary phase and consequently reduce the separation efficiency [29,30]. In the present study, the settling time of the solvent system (3:2:5, *v*/*v*/*v*) ethyl acetate/butanol/ultrapure water) was approximately 15 s and complied with the proposed ideal conditions. A short elution time depends on the partition coefficient k, which is defined as the ratio of the solute concentration between the two equilibrated immiscible solvent phases. The ratio is calculated as absorbance of upper phase divided by absorbance of the lower phase (k_U/L_) [29] The k-value of the crude synthesis extract, containing non-esterified xanthylium derivatives only, was determined by measuring the absorbance of upper and lower phase at 440 nm after shaking the solvent system to estimate the effect of co-extracted compounds. The k-value of the chosen CCC system was calculated as 2.15, which is near recommended k-values 1.0 ≤ k_U/L_ ≤ 2.0 for the tail-to-head mode [29]. For the xanthylium cations isolated via HPCCC a chromatographic purity determined at 280 nm of 83.9% was reached. Additionally, colorless carboxymethine-linked catechin dimers were present in the fraction. The formation reaction of xanthylium derivatives has diverse intermediate stages [18,19], which include the formation of numerous by-products. It can be assumed that the reaction of carboxymethine-linked catechin dimers with an additional glyoxylic acid before dehydration and resulting ring closure to a xanthene might be possible. Also, the presence of xanthene-xanthylium trimers, xanthylium quinones or xanthylium lactones [5,9,17] might be assumed even if they were below the limit of detection in the present study. The results imply that HPCCC is a promising approach for the isolation of xanthylium derivatives. The fraction of non-esterified xanthylium cations was used as standard (calculated as NJ2 equivalents) and for the investigation of the color properties (2.2. and 2.3.) in white wine. The application of the HPCCC protocol for the isolation of the ethyl esters of xanthylium cations yielded only in mixed fractions and was therefore not applied for the isolation of the esterified derivatives. The esters were isolated via SPE described as follows.

#### 2.1.3. Solid Phase Extraction (SPE)

By application of SPE, xanthylium derivatives could successfully be separated from other compounds present in the reaction mixture. The application was based on the approach of George et al. [23]. The xanthylium cations eluted directly after sample loading and the xanthylium cation ethyl esters during the fifth washing step with an ethanol concentration of 40%. A chromatographic purity (at 280 nm) of 82.9% and 76.1% was achieved for the xanthylium cations and the corresponding ethyl esters, respectively. Further compounds in the isolated fractions are traces of the respective other compound class and traces of by-products of the formation reaction, predominantly colorless compounds like carboxymethine-linked catechin dimers. Lyophilized fractions from the isolation via SPE were used for the investigation of the color properties (ethyl esters only) and for the evaluation of the stability in wine.

### 2.2. Impact on Color Parameters (CIELab)

The xanthylium derivative fractions obtained by HPCCC and SPE were dissolved in a young Riesling wine at different concentrations (0.1, 0.5, 1.0, 2.5 and 5.0 mg/L) to determine their color properties. No xanthylium derived pigments were detected by UHPLC-DAD-ESI-MS^n^ prior to xanthylium derivative addition. In the CIELab color system, the color is described by the five parameters L*, a*, b*, h*, and C*. L* represents the lightness of the color, a* describes the green/red part of the color (a* < 0 green, a* > 0 red), b* the yellow/blue part (b* > 0 yellow, b* < 0 blue), h* the tone (hue) of color and C* is the chromaticity (Chroma). Table 2 displays the color parameters h*, C* and ΔE* of the untreated and spiked Riesling wines.

**Figure 4:**
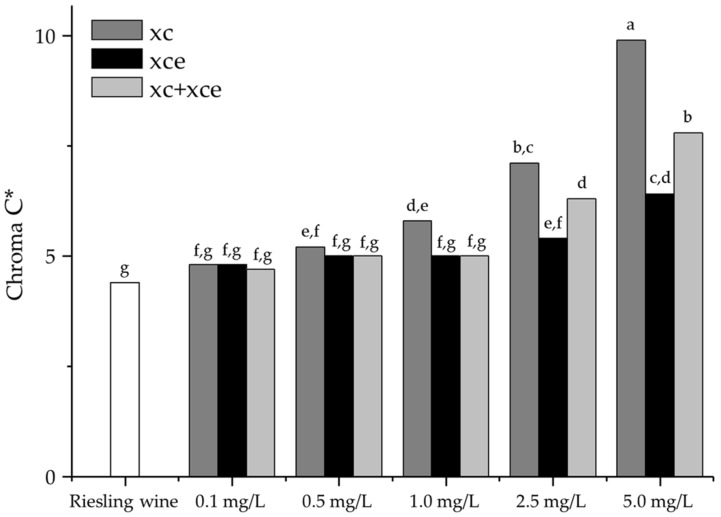
Color parameter C* of Riesling wine and spiked wines. xc = non-esterified xanthylium cations, xce = xanthylium cation ethyl esters, xc + xce = equivalent mixture of xanthylium cations, and its corresponding ethyl esters (1:1, *w*/*w*). Bars with different letters are significantly different at *p* ≤ 0.05 (*n* = 2; mean standard deviation 3%).

The differences in lightness, chromaticity, and overall color between the genuine and the spiked Riesling were low and the color parameters were similar. The low concentrations of xanthylium derivatives are reflected by these low changes of the parameters. Lightness (L*) was not affected by the addition of the compounds. The a*-value and b*-value reflect the given color impression of the samples corresponding with the yellow to orange color of the pigments. The hue reflects the natural color properties of the different xanthylium derivatives. The observed slight shift of the h*-value from approximately 100° (greenish-yellow) to 90° (yellow) conforms with the yellow to orange color appearance of the different pigments. The development of the color intensity is described by the C*-value. Besides changes in the hue, the color intensity is also increased. Figure 4 demonstrates the different changes of the color intensity related to compound class.

It needs be mentioned that the non-esterified cations had a stronger impact on the color intensity, the reason remaining unclear. The Delta E (ΔE*) value is used to express the overall color difference between a sample and the control. Values above 1 reflect a difference which is noticeable for a trained observer and values above 2 are clearly perceived [31]. The lower concentrations provoked only slight differences regardless of the compound. An addition of 2.5 mg/L of xanthylium cations caused an observable color difference (ΔE* = 2.82), whereas the addition of the same amount of xanthylium cation ethyl esters might only be observable by trained sensory assessors (ΔE* = 1.42). The addition of the corresponding concentration of the mixture of both compounds led to a ΔE* of 2.08. This suggests a higher coloring potential of the non-esterified xanthylium cations compared to the ethyl esters. The ΔE* value of the mixture conformed to the expectations. The mixture showed similar effects as the non-esterified cations respectively esters, which implies that the cations and the ethyl esters show no marked interactive effects. By the addition of xanthylium derivatives, the wine color developed from light greenish-yellow to yellow-brown. This alteration conforms to changes of wine color that can be observed during wine aging [32]. Therefore, xanthylium derivatives might have a major impact on the development of color defects and browning processes.

### 2.3. Sensory Evaluation of The Impact of Xanthylium Derivatives

**Figure 5:**
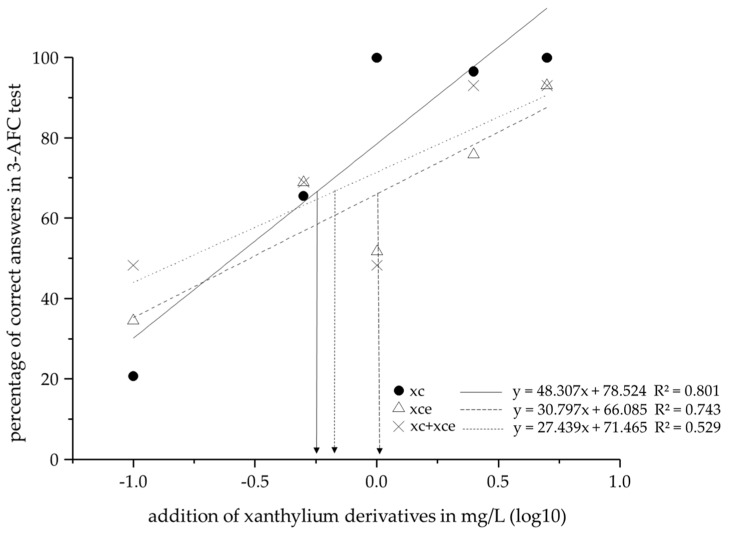
Correlation between added amount of xanthylium derivatives and correct answers in a 3-AFC test, comparing non-esterified xanthylium cations (xc), xanthylium cation ethyl esters (xce) and the mixture of both compound classes (xc + xce). The arrows indicate the respective perception threshold.

On the basis of a 3-AFC test, the perception threshold of xanthylium cations, the corresponding ethyl esters, and a 1:1 (*w*/*w*) mixture of both compounds was investigated. The threshold of the non-esterified cations was calculated as 0.57 mg/L, for the ethyl esters as 1.04 mg/L and for the mixture as 0.67 mg/L according to ISO 13301:2002 [33]. The differences in perception threshold might imply synergistic or counteractive effects of the compounds like the anti-copigmentation effect as described by Rustioni et al. for anthocyanins and grape polyphenols [34]. However, a separate study on copigmentation did not support this assumption. For the assessment of copigmentation effects xanthylium cations have been mixed with the cofactors xanthylium cations (10−200 mg/L), (+)-catechin, or quercetin (10−100 mg/L) at a one-to-one molar ratio of the compounds in 50% aqueous ethanol. In all mixtures no enhancement or shift of the absorbance maximum at 440 nm after 30 min was observable (data not shown). The correlation between the added amount of xanthylium derivatives and the percentage of correct answers in the sensory analysis are plotted in Figure 5.

A total of 75% or more correct answers is required to determine the sensory threshold. This requirement was met for all three sample sets and the threshold was determined at 66.67%. The non-esterified xanthylium cations showed the lowest perception threshold substantiated by a high correlation coefficient (R^2^ = 0.801). The perception threshold of the corresponding ethyl esters is approximately two times higher, whereas the visual detection threshold of the mixture was only slightly higher compared to the non-esterified xanthylium cations. This underlines the assumption that the non-esterified cations show a higher chromaticity in comparison to their corresponding ethyl esters. The results are in accordance with the observations of the ΔE*. The thresholds were expectedly correlated with the Chroma C* but showed no coherence with the h*-value. This shows that the Δ*E* value was mainly based on the change of the chromaticity, whereby the slight shift of the hue (see Table 2) was in accordance with the absence of copigmentation effects. A considerable increase in Chroma C* was observable above the perception threshold (non-esterified cations between 0.5 and 1.0 mg/L, esters between 1.0 and 2.5 mg/L). Moreover, non-esterified xanthylium cations showed the lowest threshold and the highest increase in color intensity. The perception threshold of the analyzed pigments is comparatively low. Other abundant polyphenolic pigments like anthocyanins show perception thresholds between 0.7 and 5.7 mg/L [35], revealing a high potential of xanthylium derivatives as color active compounds.

### 2.4. Concentration in Commercial Wines

Because of their low perception threshold, the concentrations of non-esterified xanthylium cations and xanthylium cation ethyl esters were quantified in commercial wines using UHPLC-DAD-ESI-MS (SRM). The analysis of the isolated fractions confirmed that the non-esterified cations elute between 8.4 and 11.0 min and the ethyl esters between 12.5 and 14.0 min. Xanthylium cations generally produce product ions at *m/z* 617. The [M]^+^ of the esterified compounds is *m/z* 645 [22]. Both species show a specific mass transition due to the Retro-Diels-Alder fission of the C-ring of the catechin moiety. This leads to a loss of 152 Da with fragment ions at *m/z* 465 and *m/z* 493, respectively [25]. This transition was used for the quantification of xanthylium derivatives in wines. The results of the screening disclosed that none of the described compounds can be detected above the limit of detection in the analyzed wines. However, 25 wines, including 17 white wine samples and 8 rosé wine samples, contained trace amounts of compounds with the same fragmentation pattern in the elution window of non-esterified xanthylium cations. Table 3 summarizes the result of the UPHLC-DAD-ESI-SRM analysis.

**Figure 6:**
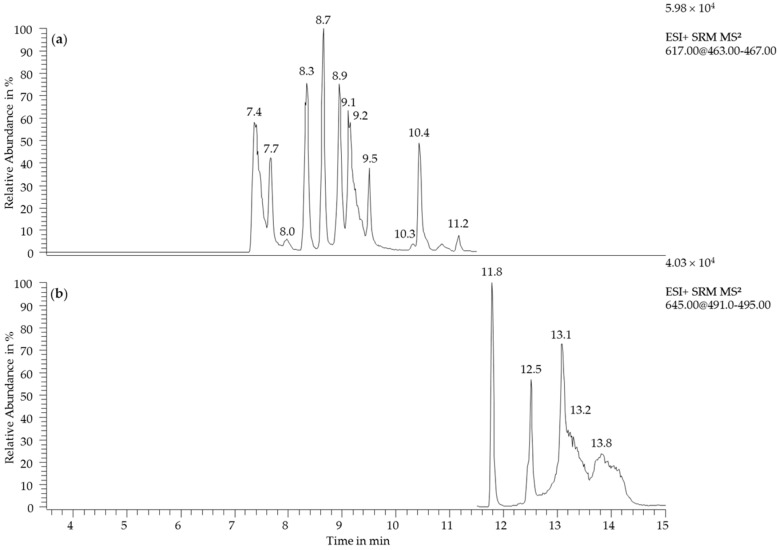
UHPLC-ESI^+^-MS (SRM) chromatogram of the synthesis medium with catechin and epicatechin after incubation at 45 °C for 6 days. (**a**) Single reaction monitoring for xanthylium cations *m/z* 617 → 465; (**b**) Single reaction monitoring for xanthylium cation ethyl esters *m/z* 645 → 493. Peak labels indicate retention time.

These compounds were detected in traces with concentrations below 0.1 mg/L and were thus under the perception threshold according to the results of the 3-AFC test. Wine type, grape variety, origin, or vintage did not have an influence on the appearance of xanthylium-like compounds. The detected peaks can be partly assigned to the described derivatives of (+)-catechin. Non-esterified xanthylium cations 3 (t_R_ = 9.2 min) and 5 (t_R_ = 11.0 min) have been detected in the synthesis medium and in the wine samples. (+)-Catechin may form up to six different xanthylium cations and corresponding ethyl esters, respectively [18], and besides catechin, (−)-epicatechin is also available for the formation of xanthylium derivatives [24,25]. The epicatechin derived xanthylium derivatives lead to a higher absorbance at 440 nm [25]. Wine contains both isomers and the formation of xanthylium derivatives underlies a greater variation than in the model wine system which contained catechin only. This was confirmed with a xanthylium derivative synthesis with catechin and epicatechin under the same conditions as applied for the isolation. Figure 6 displays the separation of the resulting derivatives via LC-MS.

In comparison to the synthesis with catechin as described in Section 2.1, a minimum of 12 non-esterified xanthylium cations was detected. Also, a diversification of the ethyl esters formed was observed by an amplification of the chromatographic hump between 13 and 14 min. The peaks (t_R_ = 7.4, 7.5, 7.7, 7.9, 8.3, 8.7, 9.1, 10.3, 10.4, 10.9, 11.3 min) detected in the wine samples were assigned to catechin-epicatechin-derived xanthylium derivatives. The color of rosé wines was characterized by the anthocyanin content that was between 10–77 mg/L calculated as malvidin equivalents. A peak with a retention time of 3.9 min showed the same ion of the particular mass selected as the xanthylium cations (*m/z* 617 → 465). The loss of 152 Da is specific for xanthylium derivatives but also for other catechin derived compounds due to Retro-Diels-Alder fragmentation. The unknown compound was found in 41 wine samples, including all 12 rosé wines. Its occurrence was not related to grape variety, origin, or vintage but correlated with the appearance of trace amounts of xanthylium cations. This concomitant appearance of the compounds was observed in 19 out of the 25 samples. Since all 12 rosé wines contained the unknown compound and eight of them additionally contained traces of xanthylium cations, a prevalence may be assumed for red grape varieties. It might be supposed that the compound contains catechin or xanthylium derived structures that leads to a signal in the SRM mode. The correlated occurrence of the compound and xanthylium cations implies that it might be a reaction product of the xanthylium derivatives. Due to its low concentration, a structural elucidation was not possible and needs further investigation.

### 2.5. Storage Stability in Wine

**Figure 7:**
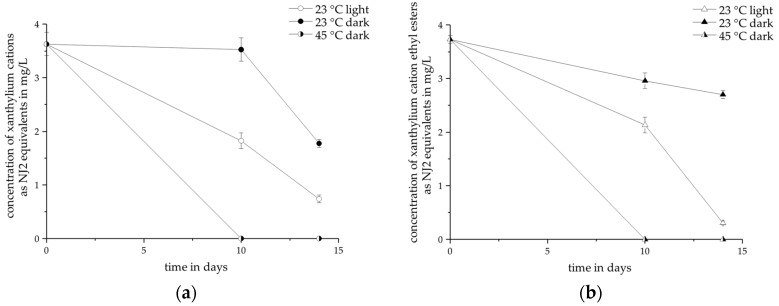
Development of the concentration in white wine under three different storage conditions over a period of 14 days (*n* = 2), quantified at 440 nm as NJ2 equivalents (**a**) non-esterified xanthylium cations; (**b**) xanthylium cation ethyl esters.

The investigation of the stability of xanthylium derivatives in white wine revealed information on the impact of xanthylium derivatives on the color and aging-related color changes in white wine. The storage stability of xanthylium derivatives depended on light exposure and temperature. Figure 7 shows the decrease in concentration of non-esterified xanthylium cations (a) and xanthylium cation ethyl esters (b) over a period of two weeks.

At a temperature of 45 °C, the added xanthylium cations and the xanthylium cation ethyl esters were degraded after ten days. Light exposure accelerated the degradation of the compounds during storage at room temperature (23 °C). Xanthylium derivatives are known to be prone to light-induced degradation [23,24]. However, also without light exposure, the compounds were degraded, the degradation rate being much higher at 45 °C. The thermal instability of the compounds supported the assumption that the xanthylium derivatives are simultaneously formed and degraded in the synthesis medium. Xanthylium cations and the esters displayed the same degradation rate but the esters proved to be more stable at 23 °C in the dark after two weeks. The depletion of the compounds seemed to be generally delayed by the esterification. The results of the stability tests imply that xanthylium derivatives undergo structural changes in wine. These changes might be provoked by the presence of caffeic acid which reduces the stability of xanthylium derivatives [23]. A recent study demonstrated epicatechin-based polymers containing a xanthylium derivative illustrating evidence for subsequent reactions of xanthylium derivatives [36]. The spiked wines remained perceptible yellow colored but the MS data revealed no information on the identity of newly formed pigments. It can be expected that the evolving pigments possess a molecular weight above 2000 Da and were therefore not detected by the MS. The assessment of the color parameters (see Table 4) showed that the development of the color parameters depended on the storage conditions. The color of the control sample showed considerable color changes at 45 °C and the changes of the spiked wines were comparable. This suggests that the color changes of both wines (blank and spiked) was dominated by competing browning reactions which do not necessarily include xanthylium derivatives. There were no perceptible changes at 23 °C. This might support the assumption of the formation of other pigments.

Xanthylium cations and their corresponding ethyl esters were not detected at concentrations above the detection threshold in bottled wines. However, xanthylium derivatives can be considered as intermediate products in oxidative processes that potentially lead to browning. Thus, this compound class still has a potential influence on color development in wines. Xanthylium derivatives as oxidation products might play a role in young wines or musts and are depleted in old wines during storage. Particularly high storage temperatures and light exposure influence the stability of the pigments [23,24].

## 3. Materials and Methods 

### 3.1. Synthesis and Isolation of Xanthylium Derivatives

Xanthylium derivatives were synthesized by the reaction of (+)-catechin and glyoxylic acid under oxidative conditions in a wine-like model solution. A total of 500 mg/L (+)-catechin (added as (+)-catechin monohydrate (>98%, Sigma-Aldrich GmbH, Steinheim, Germany) was added to a model wine solution. The model wine solution was prepared by adding 0.011 M potassium bitartrate (added as dipotassium L(+)-tartrate hemihydrate p. a. ≥99%, Fluka Chemie AG, Buchs, Switzerland)) and 0.007 M L(+)-tartaric acid (Oestreich GmbH, Appenweier, Germany) to 5 L 12% aqueous ethanol (≥99.9%, Th. Geyer GmbH & Co. KG, Renningen, Germany). For the synthesis of non-esterified xanthylium cations, all compounds were additionally dissolved in 1 L water. The solution was treated in the ultrasonic bath for 10 min and subsequently stirred for 24 h. The pH 3.2 ± 0.1 was adjusted by the addition of 1 M sodium hydroxide solution (Acros Organics N.V., Geel, Belgium) or 1 M hydrochloric acid (32%, Carl Roth GmbH & Co. KG, Karlsruhe, Germany) [21,23]. In addition, 0.60 mg/L copper(II) (added as copper(II) sulfate pentahydrate >99%, AppliChem GmbH, Darmstadt, Germany) and 1.50 mg/L iron(II) (added as iron(II) sulfate heptahydrate 99.5%, Merck KgaA, Darmstadt, Germany) were supplemented. Glyoxylic acid was added in a concentration of 0.001 M (added as 50% solution in H_2_O, Merck KgaA) to achieve a molar ratio of catechin to glyoxylic acid of 2:1. The reaction mixture was incubated at 45 °C for a period of 15 days in the dark. The formation of derivatives was monitored by UHPLC-DAD-ESI-MS^n^. After incubation, the reaction mixture was cooled to 7 °C and used for SPE directly. Catechin and non-colored intermediates were quantified as (+)-catechin equivalents at 280 nm (1.0−600 mg/L) and xanthylium derivatives as NJ2 equivalents at 440 nm (0.1–100 mg/L) by external calibration. The calibration curves showed a regression of R² = 0.999 and 0.998. For HPCCC separation, the sample without ethanol was lyophilized to obtain a xanthylium cation fraction free of any esterified compounds. The isolation was performed by solid-phase extraction according to the method described by George et al. [23] and by countercurrent chromatography according to the recommendations by Ito [29], respectively.

#### 3.1.1. UHPLC-DAD-ESI-MS^n^ Identification of Xanthylium Derivatives

The identification and quantification of xanthylium derivatives in the synthetic medium were conducted on an Acquity I-Class system (Waters, Milford, MA, USA) coupled with an LTQ-XL ion trap mass spectrometer (Thermo Scientific Inc., Dreieich, Germany). The column was a Waters HSS T3 C-18 1.8 μm particle size (2.1 × 150 mm) equipped with a security guard cartridge of the same material (2.1 × 5 mm, 1.8 μm) hold at 40 °C. The following gradient was used at a flow rate of 0.4 mL/min: 0 min 98% A; 20 min 60% A; 21 min 0% A; 25 min 0% A; 26 min 98% A; 30 min 98% A. Eluent A was water/formic acid (99.9/0.1, *v*/*v*) and eluent B acetonitrile/formic acid (99.9/0.1, *v*/*v*). The injection volume was 5 μL. The conditions of the mass spectrometer were as follows: capillary was set at a temperature of 350 °C in positive electrospray ionization (ESI) mode and was operated at a voltage of 14.0 V. The source voltage was maintained at 0.0 kV at a current of 100 μA. The tube lens was adjusted to 55.0 V. Nitrogen was used as the sheath, auxiliary, and sweep gas at a flow of 60, 8, and 1 arbitrary units, respectively. Collision-induced dissociation spectra were obtained at 35 eV using helium as the collision gas. The full scan mode was used with a range of 250 to 2000 Da. 

#### 3.1.2. High-performance Countercurrent Chromatography (HPCCC)

The CCC was performed using a high-performance countercurrent chromatograph model DE Spectrum Centrifuge (Dynamic Extractions, Tredegar, UK). The preparative coil consists of PTFE tubes with an inner diameter of 1.6 mm and a total volume of 136 mL. The HPCCC system was equipped with a Blue Shadow 40P solvent delivery pump (Knauer, Berlin, Germany), a Blue Shadow D50 UV–vis detector (Knauer), a Foxy R1 fraction collector (Teledyne ISCO, Lincoln, NE, USA), a Degasys DG-1210 degasser (Uniflows, Tokio, Japan) and a recirculating chiller F-108 (Büchi, Essen, Germany). The selection of the two-phase solvent system for the target compounds is the most important step in CCC. Due to its polar character, a biphasic solvent system consisting of ethyl acetate/n-butanol/water (3:2:5, *v*/*v*/*v*) was used. The k-value was calculated according to the equation k = E_upper phase_/E_lower phase_ [29]. The determination of the k-value was performed by adding 0.5 mg of the sample to both mutually equilibrated solvent phases (10 mL). For equilibration, the sample was thoroughly mixed, and the distribution of the sample constituents in both phases was determined at 440 nm (Genesys 6, Thermo Fisher Scientific GmbH, Dreieich, Germany). The separation was carried out at a revolution speed of 1600 rpm and a flow rate of 6 mL/min. The upper organic phase was used as the mobile phase in the tail-to-head elution mode. The injection valve was equipped with a 6 mL sample loop. The sample solutions were prepared by dissolving 500 mg of the lyophilized synthesis reaction mixture in a mixture of upper and lower phase (8 mL 1:1, *v*/*v*). Chromatographic separations were monitored at 280, 440 and 460 nm. Fractions were collected at intervals of 30 s.

#### 3.1.3. Solid-phase Extraction (SPE)

SPE was carried out using multimode cartridges CHROMABond C18 ec (Macherey-Nagel GmbH & Co. KG, Düren, Germany) consisting of an octadecyl-modified silica gel with a bed volume of 10 g. The method is based on the extraction procedure of George et al. [23]. The cartridges were conditioned with 120 mL ethanol and then equilibrated with 120 mL ultrapure water obtained from a Synergy purification system (Millipore, Molsheim, France). The sample (30 mL) was loaded onto the cartridges. The xanthylium cations were collected during this step. The cartridges were then washed with 120 mL of water, followed sequentially by 120 mL of 5% and 7.5% aqueous ethanol solutions, and 70 mL of 25% aqueous ethanol solution. The ethyl esters of the xanthylium cations were then eluted with 50 mL of 40% ethanol. The solvents were evaporated after SPE and the residue was subsequently lyophilized.

### 3.2. Impact On Color Parameters (CIELab)

For the evaluation of the influence of different xanthylium derivative concentrations, 2.62 mg of xanthylium cations (purity approximately 82.9%) and 1.08 mg of xanthylium cation ethyl esters (purity approximately 76.1%) were dissolved in 65.82 and 24.91 mL of a 2015 Riesling wine. These stock solutions were diluted to an addition of 0.1, 0.5, 1.0, 2.5 and 5.0 mg/L xanthylium derivatives, respectively. The blind samples contained no xanthylium derivatives. The absorbance spectra were measured with a spectrophotometer, using a 1 cm path length cell. Measurements were taken every 1 nm between 220 and 780 nm. Ultrapure water was used as the blank. From the spectra, the rectangular coordinates L* a* b* and the cylindrical coordinates CIE C* and h* were calculated using CIE method [37], and Delta E (ΔE*) according to ISO 11664-4:2008 [31]. For statistical analysis the XLSTAT software was used. An Analysis of Variance (ANOVA) and separation of mean by Tukey was performed. The level of significance was defined as *p* ≤ 0.05.

### 3.3. Sensory Evaluation of The Impact of Xanthylium Derivatives

Aliquots of 1.0 mL of each wine sample prepared as described in Section 3.2. were transferred into blank glass vials. A panel of 29 judges experienced in sensory analysis performed the 3-AFC tests by visual inspection only. In each triangle, one sample contained xanthylium derivatives and the panelist had to select the most intensively colored sample by forced choice principle [38]. The threshold was defined as the concentration with a probability of detection of distinction at 0.5 (50%). The probability of correct answer (33%) was included in the calculation. Based on correlation diagrams, the logarithmic concentration was read off at a proportion of 66.67% of correct answers. The respective threshold in mg/L was determined by calculation of an exponential function [37].

### 3.4. Concentration in Commercial Wines

#### 3.4.1. Wines

The concentration of xanthylium derivatives was determined in 70 wines, 58 white wines, and 12 rosé wines. The wines were from Europe, America, Australia, and Africa. The sample set contained 16 different grape varieties (no cuvée) and nine vintages. The full sample list is given in Table 5.

#### 3.4.2. UHPLC-DAD-ESI-MS Selected Reaction Monitoring (SRM)

The quantification of xanthylium derivatives in the wines was carried out using a UHPLC-DAD-ESI-MS system as defined in Section 3.1.2. Gradient program and the conditions of the mass spectrometer were identical. Deviating from the method outlined above the selected reaction monitoring was used. Two transitions were analyzed in two timeframes. From min 1.5 to 11.5 the fragmentation at *m/z* 617 to 465 and between min 11.5 to 20 the fragmentation at *m/z* 645 to 493 was analyzed. The injection volume was reduced to 3 μL. The xanthylium derivatives were quantified as NJ2 equivalents by external calibration (0.1−100 mg/L) based on the SRM signal. The calibration curve showed a regression of R² = 0.996.

### 3.5. Storage Stability in Wine

For the evaluation of the storage stability of the xanthylium derivatives, 0.3 mg of xanthylium cations or xanthylium cation ethyl esters was dissolved in 65 mL of a 2015 Riesling wine (see Section 3.2 and Section 3.3) and sonicated for 10 min in an ultrasonic bath, yielding an initial concentration of about 3.8 mg/L, which is approximately five times higher than the average value of the perception thresholds. Aliquots of 10 mL were stored under different temperatures and light conditions summarized in Table 6. The distance of the light source to the sample was 30 cm. The experiments were carried out in duplicate.

The concentration of the sample solution was measured via UHPLC-DAD-ESI-MS^n^ as described in Section 3.1.1. at three points within two weeks (day = 0, 10 and 14). Moreover, the absorbance spectra for the assessment of the color parameters of the wine spiked with non-esterified xanthylium cations stored under certain conditions were determined as described in Section 3.2.

## 4. Conclusions

The study revealed new aspects regarding the impact of xanthylium derivatives on the color of white wine. The sensory evaluation demonstrated the extraordinarily low perception thresholds of the compounds in young Riesling wine. However, due to their low concentration in wine, which might be a result of their low stability and high reactivity, non-esterified xanthylium cations and their ethyl esters do not seem to have a direct impact on white wine color. The analysis of the color parameters, especially the Delta E value, showed that the compounds led to a perceptible change of wine color at very low concentrations, whereby the non-esterified cations induced greater alterations. The results presented here clearly demonstrate that xanthylium derivatives play only a secondary role in white wine color. Xanthylium derivatives might be considered as intermediate products that react to more complex compounds, but other yet unknown reactions might also play a considerable role in color formation. The study underlined the complexity of the color evolution in white wine and a possible effect of xanthylium derivatives regarding alterations that occur by polymerization during wine aging.

## Figures and Tables

**Table 1:** Summary of UHPLC-DAD-ESI-MS^n^ data of (+)-catechin, intermediates and formed xanthylium derivatives detected in the reaction solution. The numbers beyond the compounds in the last column indicate the elution order within one species. cml = carboxymethine-linked, xc = xanthylium cation.

No.	t_R_ MS (min)	Specific UV λ_max_	[M + H]^+^ (*m/z*)	Fragment ions MS² (*m/z*)	Fragment ions MS³ (*m/z*) @ xyz MS²	Compound
1	5.6	281	637	485, 347, 467, 619, 333	333, 467, 291, 345	cml (+)-catechin dimer 1
2	6.5	280	291	123, 139, 165, 273, 151	123	(+)-catechin
3	7.8	^1^	637	347, 485, 329, 467, 291	329, 123, 311, 195	cml (+)-catechin dimer 2
4	8.0	^1^	637	347, 485, 329, 467, 291	329, 123, 311, 195	cml (+)-catechin dimer 3
5	8.4	441	617 ^2^	465, 599	421, 447, 313	xanthylium cation 1
6	8.9	440	617 ^2^	465, 599	313, 447, 421	xanthylium cation 2
7	9.2	441	617 ^2^	465, 599	447, 421, 313	xanthylium cation 3
8	10.6	440	617	465, 599	447, 421, 313	xanthylium cation 4
9	10.8	281	619	467, 327, 449, 291, 583	315, 449, 327	xanthylium lactone
10	11.0	441	617 ^2^	465, 599	421, 447, 313	xanthylium cation 5
11	11.8	462	645 ²	493, 599, 341	493, 341	xc ethyl ester 1
12	12.5	462	645 ^2^	493, 599, 341	447, 341, 295	xc ethyl ester 2
13	13.1	462	645 ^2^	493, 599, 341	341, 447	xc ethyl ester 3
14	14.0	462	645 ^2^	493, 341, 599	447, 341, 295	xc ethyl ester 4

^1^ Not detectable at UV spectrum. ^2^ [M]^+^.

**Table 2:** Selection of CIELab parameters hue angle (h*), chromaticity (C*) and color difference (ΔE*) of untreated and spiked wines. xc = non-esterified xanthylium cations, xce = xanthylium cation ethyl esters, xc + xce = 1:1 (*w*/*w*) mixture of both compounds. The numbers indicate the addition of compound in mg/L. Values with different letters are significantly different at *p* ≤ 0.05 (*n* = 2; mean standard deviation 3%).

Sample	CIELab Parameters		
	h*	C*	ΔE*
Riesling wine	102.0 ^a^	4.4 ^g^	
0.1 xc	101.8 ^a^	4.8 ^f,g^	1.36 ^f,g^
0.5 xc	101.6 ^a^	5.2 ^e,f^	1.45 ^f^
1.0 xc	102.3 ^a^	5.8 ^d,e^	1.75 ^e^
2.5 xc	102.2 ^a^	7.1 ^b,c^	2.82 ^c^
5.0 xc	99.9 ^a^	9.9 ^a^	5.49 ^a^
0.1 xce	101.2 ^a^	4.8 ^f,g^	1.26 ^f,g^
0.5 xce	100.6 ^a^	5.0 ^f,g^	1.34 ^f,g^
1.0 xce	102.3 ^a^	5.0 ^f,g^	1.10 ^g^
2.5 xce	99.9 ^a^	5.4 ^e,f^	1.49 ^e,f^
5.0 xce	96.9 ^a^	6.4 ^c,d^	2.16 ^d^
0.1 xc + xce	101.5 ^a^	4.7 ^f,g^	1.24 ^f,g^
0.5 xc + xce	101.3 ^a^	5.0 ^f,g^	1.35 ^f,g^
1.0 xc + xce	102.3 ^a^	5.0 ^f,g^	1.10 ^d^
2.5 xc + xce	100.8 ^a^	6.3 ^d^	2.08 ^d^
5.0 xc + xce	98.6 ^a^	7.8 ^b^	3.48 ^b^

**Table 3:** Wine samples containing traces of xanthylium derivatives. Peaks that are assignable to compounds identified in the synthesis medium based on their retention time (t_R_) are underlined. The concentration was calculated as sum of all detectable peaks with a mass transition of *m/z* 617 to 465 in each sample. Origin Country Codes in alphabetical order: A = Austria, ARG = Argentina, D = Germany, E = Spain, F = France, I = Italy, RCH = Chile, SLO = Slovenia, USA = United States, ZA = South Africa.

No.	Grape Variety	Origin Country Code (Vintage)	t_R_ (min) *m/z* 617 → 465	NJ2 Equivalents (mg/L)
W4	Chardonnay	A (2015)	7.9	< 0.1
W8	Chardonnay	D (2015)	7.9	< 0.1
W9	Chardonnay	D (2015)	7.7, 7.9	< 0.1
W25	Macabeo	E (2015)	7.9	< 0.1
W27	Pinot Gris	D (2012)	9.1, 9.2	< 0.1
W29	Pinot Gris	D (2015)	8.3, 8.7, 10.4	< 0.1
W32	Riesling	D (2008)	8.7, 9.2	< 0.1
W35	Riesling	D (2014)	7.9, 8.3	< 0.1
W36	Riesling	D (2015)	7.9, 8.7, 10.3	< 0.1
W43	Sauvignon Blanc	E (2015)	7.9	< 0.1
W44	Sauvignon Blanc	E (2015)	7.4	< 0.1
W46	Sauvignon Blanc	F (2015)	7.9, 8.7, 10.4	< 0.1
W47	Sauvignon Blanc	F (2015)	7.9	< 0.1
W48	Sauvignon Blanc	I (2014)	7.9, 8.7, 10.3	< 0.1
W51	Sauvignon Blanc	RCH (2016)	7.9	< 0.1
W52	Sauvignon Blanc	SLO (2015)	7.9	< 0.1
W54	Sauvignon Blanc	ZA (2016)	7.9	< 0.1
W59	Dornfelder	D (2015)	7.5, 9.1, 9.8, 10.9	< 0.1
W60	Grenache	E (2015)	7.9	< 0.1
W61	Grenache	USA (2015)	9.1, 11.0	< 0.1
W62	Malbec	ARG (2015)	7.9, 11.0	< 0.1
W63	Malbec	USA (2015)	7.5, 9.1, 9.8, 11.0, 11.3	< 0.1
W64	Pinot Noir	D (2015)	7.4, 7.9, 10.4, 11.0	< 0.1
W65	Pinot Noir	D (2015)	7.5, 7.9, 9.1, 9.8, 11.0	< 0.1
W67	Shiraz	F (2015)	7.5, 8.3, 9.1, 11.0	< 0.1

**Table 4:** Selection of CIELab parameters lightness (L*), color opponents green/red (a*) and yellow/blue (b*), and color difference (ΔE*) of untreated and with non-esterified xanthylium cations (xc) spiked Riesling wine stored under three different storage conditions at beginning of the experiment and after a period of 14 days (*n* = 2).

Sample	CIELab parameters			
	L*	a*	b*	ΔE* ^1^
Riesling wine (blank test) t_0_	98.7 ± 0.6	−1.0 ± 0.1	4.9 ± 0.1	
Riesling wine, 23 °C light t_14_	98.8 ± 0.0	−0.7 ± 0.0	4.5 ± 0.0	0.47
Riesling wine, 23 °C dark t_14_	98.8 ± 0.0	−1.0 ± 0.0	5.2 ± 0.1	0.37
Riesling wine, 45 °C dark t_14_	97.6 ± 0.0	−1.3 ± 0.1	10.3 ± 0.0	5.59
xc in Riesling wine t_0_	97.9 ± 0.8	−1.5 ± 0.1	9.2 ± 0.0	
xc, 23 °C light t_14_	98.1 ± 0.0	−0.9 ± 0.0	8.0 ± 0.0	0.59
xc, 23 °C dark t_14_	98. 2± 0.1	−1.3 ± 0.0	8.7 ± 0.1	1.30
xc, 45 °C dark t_14_	96.7 ± 0.0	−1.0 ± 0.0	13.6 ± 0.0	4.59

^1^ Calculated as the difference in-between blanks and spiked wine samples.

**Table 5:** Overview of wines used for screening, in alphabetical order referred to grape variety and origin country, respectively. All wines were analyzed in 2017.

No.	Grape Variety	Origin Country Code (Number; Vintage^x share of number^)
W1	Albarino	E (1; 2015)
W2, W3	Blanc de Noir	D (2; 2007, 2015)
W4–W21	Chardonnay	A (2; 2015, 2016) CRO (1; 2015) D (12; 2015^×5^, 2016^×7^) I (1; 2015) RO (1; 2013) USA (1; 2015)
W22	Chenin Blanc	ZA (1; 2016)
W23	Colombard	F (1; 2010)
W24, W25	Macabeo	E (2; 2015)
W26–W29	Pinot Gris	D (4; 2007, 2012, 2015^×2^)
W30–W39	Riesling	D (9; 2007^×2^, 2008, 2010, 2014^×2^, 2015^×3^) H (1; 2005)
W40–W54	Sauvignon Blanc	ARG (1; 2014) D (1; 2013) E (3; 2015) F (3; 2012, 2015^×2^) I (1; 2014) NZ (1; 2016) RCH (2; 2014, 2016) SLO (1; 2015) ZA (2; 2016)
W55–W58	Verdejo	E (4; 2015)
W59	Dornfelder	D (1; 2015)
W60, W61	Grenache	E (1; 2015) USA (1; 2015)
W62, W63	Malbec	ARG (1, 2015) USA (1; 2015)
W64–W66	Pinot Noir	D (2; 2015) F (1; 2016)
W67–W69	Shiraz	F (3; 2015^×2^, 2016)
W70	Tempranillo	E (1; 2015)

**Table 6:** Storage conditions for the investigation of the storage stability of xanthylium derivatives in white wine (2015 Riesling wine), xc = xanthylium cation.

Compound	Temperature (°C)	Light Conditions
xanthylium cations	23	light ^1^
	23	dark
	45	dark
xc ethyl esters	23	light ^1^
	23	dark
	45	dark

^1^ Permanent lighting with 18 W luminescent tube (Osram Licht AG, Munich, Germany).
